# Why do multi-attribute utility instruments produce different utilities: the relative importance of the descriptive systems, scale and ‘micro-utility’ effects

**DOI:** 10.1007/s11136-015-0926-6

**Published:** 2015-01-31

**Authors:** Jeff Richardson, Angelo Iezzi, Munir A. Khan

**Affiliations:** Centre for Health Economics, Monash Business School, Monash University, Wellington Road, Clayton, VIC 3800 Australia

**Keywords:** MAU instruments, Cost-utility analysis, Utility

## Abstract

**Purpose:**

Health state utilities measured by the major multi-attribute utility instruments differ. Understanding the reasons for this is important for the choice of instrument and for research designed to reconcile these differences. This paper investigates these reasons by explaining pairwise differences between utilities derived from six multi-attribute utility instruments in terms of (1) their implicit measurement scales; (2) the structure of their descriptive systems; and (3) ‘micro-utility effects’, scale-adjusted differences attributable to their utility formula.

**Methods:**

The EQ-5D-5L, SF-6D, HUI 3, 15D and AQoL-8D were administered to 8,019 individuals. Utilities and unweighted values were calculated using each instrument. Scale effects were determined by the linear relationship between utilities, the effect of the descriptive system by comparison of scale-adjusted values and ‘micro-utility effects’ by the unexplained difference between utilities and values.

**Results:**

Overall, 66 % of the differences between utilities was attributable to the descriptive systems, 30.3 % to scale effects and 3.7 % to micro-utility effects.

**Discussion:**

Results imply that the revision of utility algorithms will not reconcile differences between instruments. The dominating importance of the descriptive system highlights the need for researchers to select the instrument most capable of describing the health states relevant for a study.

**Conclusions:**

Reconciliation of inconsistent utilities produced by different instruments must focus primarily upon the content of the descriptive system. Utility weights primarily determine the measurement scale. Other differences, attributable to utility formula, are comparatively unimportant.

## Introduction

Economic evaluation of interventions which affect health-related quality of life commonly employs cost-utility analyses (CUA) which prioritise interventions according to the cost per quality-adjusted life year (QALY). The estimation of QALYs is increasingly based upon the health state utilities predicted from a multi-attribute utility (MAU) instrument (MAUI). Each of these instruments has two components. First, the descriptive system (or classification) consists of a set of questions and response categories—items—which seek to describe a person’s health. Secondly, the utility formula (or algorithm) converts the item responses into an index of utility on a 0.00 (death)—1.00 (best health) scale.

A small number of MAUI dominate the literature. A review of articles listed on the Web of Science between 2005 and 2010 found 1,663 studies which had employed an MAUI [1]. Of these, 63 % used the EQ-5D; 15 % the HUI 2 or HUI 3; 9 % the SF-6D; and the remaining 15 % used the 15D, QWB or one of the new Assessment of Quality of Life (AQoL) instruments. The descriptive systems of these instruments, which are described in Table 1, differ significantly in size and content. Three of the instruments—EQ-5D, HUI 3 and 15D—have a preponderance of items which relate to physical health. The SF-6D has an equal number of items in the two broad domains of physical and psycho-social health, and the AQoL-8D has a preponderance of items in the psycho-social domain. Conceptually, HUI 3 has a ‘within the skin’ descriptive system: it focuses upon an individual’s body functions. The other instruments are conceptualised primarily, but not exclusively, in terms of handicap (more recently described by the WHO as activity and participation [2]), i.e. the effect of a health state on a person’s ability to function in a social environment. The items combine to describe between 3,125 and 2.4 × 10^23^ health states (EQ-5D-5L and AQoL-8D, respectively). Dissimilar descriptive systems need not result in different predicted utilities. Each of the MAUI was constructed with a common endpoint, namely the measurement of the strength of preferences for health states. These may be described in a number of ways and, in principle, each of these ways, coupled with appropriate utility weights, might produce comparable measurement. (Analogously, the weight of an object may be measured with almost identical results using scales which employ a spring, a balancing of physical weights or electronic measurement techniques.) Thus, for example, with a complete ‘within the skin’ description, individuals might envisage the consequences for their ‘activity and participation’. Similarly, brief health state descriptions might result in the same average utility as obtained from a more detailed instrument with discrepancies generated by the greater detail of the larger instrument averaging zero. In these cases, the superficially large differences in the appearance of items might mask the similarity of the instruments’ predictions.

**Table 1:** Comparison of the dimensions and content of five MAU instruments^a^

Dimension	Multi-attribute utility instruments				
	EQ-5D-5L	SF-6D	HUI 3	15D	AQoL-8D
Physical					
Physical ability/mobility/vitality/coping/control	*	*	**	**	***
Bodily function/self-care	*			***	*
Pain/discomfort	*	*	*	*	**
Senses			**	**	**
Usual activities/work	*	*		*	****
Communication			*	*	*
Psycho-social					
Sleeping				*	*
Depression/anxiety/anger	*	*	*	***	*******
General satisfaction					****
Self-esteem					**
Cognition/memory ability			*		
Social function/relationships		*			******
(Family) role		*			*
Intimacy/sexual relationships				*	*
Total items	5	6	8	15	35
Health states described^b^	3,125	18,000	972,000	3.1 × 10^10^	2.4 × 10^23^

^a^Each asterisk [*] in the table represents an item in an instrument

^b^The number of possible health states is determined by the number of items and the number of response categories per item. The EQ-5D-5L has 5 items, each with 5 response levels and therefore 5^5^=3,125 possible health states

The evidence, however, does not support this possibility. The 2005–2010 review identified 392 head-to-head comparisons of the main instruments [1]. The authors generally found a low correspondence between utilities predicted by different instruments. For example, in the three large scale surveys containing five MAUI published to date, it was found that, on average, only 56, 42 and 57 %, respectively, of the variance of one instrument could be explained by another instrument [3–5].

Each MAUI was created with the intention of employing the same scale on which 1.00 and 0.00 represent best health and death, respectively, and units quantify the desired trade-off between length and HR-QoL. Nevertheless, the range of utilities predicted by the major instruments varies from 1.59 for the EQ-5D-5L (ie −0.59 to +1.00) to 0.797 for the SF-6D [1]. This implies that the effective scales used by instruments differ and that differences in instrument utilities are, in part, explained by this.

Casual comparison cannot determine the extent to which the differences between instruments are a result of these scale effects, differences in the descriptive systems and/or differences in the preferences of people interviewed to obtain utility weights. Our review of the literature did not identify studies which analyse this question. Only one study, Whitehurst et al. [6] has compared the utilities from two instruments—the EQ-5D and SF-6D—using comparable scaling methods (DCE) to derive the utility weights. The study conclusion—that the common scaling method did not ameliorate differences in utilities, and that differences are probably attributable to the dissimilar descriptive systems—is of importance for the future direction of a research programme which seeks to reconcile the differences. It implies that research which improves the precision of utility scoring formula will not reconcile the differences. Rather, descriptive systems will need to be revised.

The aim of the present article is to further investigate the reason for the differences between predicted utilities. It does so by pairwise comparison of instrument utilities and disaggregating differences into three components: differences attributable to the two instrument scales, differences in the structure of the descriptive systems and the effect of the utility formula after taking account of the two previous effects. To avoid misleading connotations, this last amount is termed the ‘micro-utility effect’.

Methods and data used in the study are outlined below, and results presented in the following section. Their significance for the practice and future development of cost-utility analyses is then discussed. It is concluded that there is a need to refocus future developmental research to eliminate the causes of inconsistent utility measurement identified here.

## Methods and data

### Data

A multi-instrument comparison (MIC) survey was carried out in six countries: Australia, Canada, Germany, Norway, the UK and the USA. The online survey was administered by a global panel company, CINT Pty Ltd. The survey was approved by the Monash University Human Research Ethics Committee, Monash University, Melbourne, Australia, reference number CF11/3192-2011001748.

Respondents were initially asked to indicate whether they had a chronic disease and to rate their overall health on a visual analogue scale (VAS) where 0.00 represented death and 100 represented ‘best possible health’ (physical, mental and social). Quotas were then used to obtain a demographically representative sample of the ‘healthy’ public, defined by the absence of chronic disease and by a score above 70 on the VAS. Quotas were also applied to obtain a target number of respondents in each of seven chronic disease areas, viz, arthritis, asthma, cancer, depression, diabetes, hearing loss and heart disease.

Each respondent completed a total of 12 questionnaires: seven MAU instruments, three subjective well-being instruments, the ICECAP capabilities instrument, a self TTO and a VAS. Responses were subjected to a set of stringent edit procedures based upon a comparison of duplicated or similar questions and a minimum completion time. Edit procedures, the questionnaire and its administration are described in Richardson et al. [7]. Country-specific results of the edit procedures are available [8], and the database is available online [9].

For four of the instruments included in the study, utilities were calculated using algorithms provided by the instruments’ authors: SF-6D [10], HUI 3 [11], 15D [12] and AQoL-8D [13]. The 5-level EQ-5D-5L utilities were obtained from the crosswalk published by the EuroQoL Group [14], derived using methods described by van Hout et al. [15].

### Methods

**Fig. 1:**
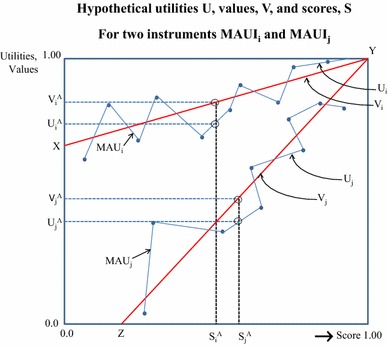
Hypothetical utilities, *U*, values, *V* and scores, *S*

The methods detailed below are illustrated in Fig. 1. This plots scores, *S*_*i*_, *S*_*j*_, derived by summing item responses from two MAU instruments, MAUI_*i*_ and MAUI_*j*_ on the horizontal axis, and the corresponding utilities, *U*, and values, *V*, on the vertical axis. Values are a linear transformation of scores and are represented by the lines *XY* and *ZY*. Due to the micro-utility effects of the MAU formula, the corresponding instrument utilities are scattered randomly around the two lines. The differing measurement scales embodied in the utility formula are illustrated by the differing slopes of *XY* and *ZY*. For a given individual, *A*, the scores from the unweighted instruments *S*_*i*_^*A*^, *S*_*j*_^*A*^ differ. Application of the two MAUI formulae result in estimates of utility which differ by (*U*_*i*_^*A*^ − *U*_*j*_^*A*^). The aim of the analysis below is to attribute this difference to a difference in the scale (*V*_*i*_^*A*^ − *V*_*j*_^*A*^), a difference in the micro-utility effect (*V*_*i*_^*A*^ *−* *U*_*i*_^*A*^) and (*V*_*j*_^*A*^ − *U*_*j*_^*A*^) and the effect attributable to the structure of the descriptive systems which results in the difference, *S*_*i*_^*A*^ *−* *S*_*j*_^*A*^.

Terminology used in the remainder of the paper is defined in Box 1.

**Box 1:** Definitions

*S*_*i*_	Unweighted score from MAU_*i*_
*U*_*i*_	Utility predicted by MAU_*i*_ using published algorithm
*U*_*j*_(*u*_*i*_)	*U*_*j*_ predicted by MAU_*j*_ rotated to the scale of *U*_*i*_ using linear transformation
*V*_*i*_	Value obtained from the score, *S*_*i*_ of MAU_i_ rotated to the scale of *U*_*i*_
*V*_*j*_(*u*_*i*_)	Value obtained from the score, *S*_*j*_, rotated to the scale of *U*_*i*_

### Measuring differences

For each respondent, absolute (sign free) differences (*U*_*i*_ *−* *U*_*j*_) were calculated for each instrument pair. (Consequently, two differences of −0.6 and +0.4 will average 0.5, not 0.1.)

### Measuring values

A two-stage method was used to calculate values, *V*_*i*_. In stage 1, the rank order of item responses were summed to obtain an initial ‘rank order’ score, *R*. For example, for the EQ-5D-5L (5 items with 5 response levels), the health state usually written as (1,1,2,2,4) would be assigned rank order numbers (5,5,4,4,2): i.e. the best response level was assigned 5.0, and the worst assigned 1.0. Consequently, *R* = 5 + 5 + 4 + 4 + 2 = 20. *R* was transformed to a (0–1) scale to obtain a score, S, using Eq. (1).

1 $$S_{i} = (R_{i} - R_{ \hbox{min} } )/(R_{ \hbox{max} } - R_{ \hbox{min} } )$$

where *R*_min_, *R*_max_ are the minimum and maximum ‘rank order’ scores which may be obtained from the instrument. In the previous example, *R* = 20, *R*_max_ = 5 × 5 = 25, *R*_min_ = 5. Therefore, *S* = (20 − 5)/(25 − 5) = 0.75. The score, *S*, defines the horizontal axis in Fig. 1.

In the second stage, scores, *S*_*i*_, were subjected to a linear transformation to obtain ‘values’ which are calibrated on the same scale as the corresponding utilities (*XY*, *ZY* in Fig. 1). To achieve this, an OLS linear regression, Eq. 2, was estimated for each instrument between utilities, *U*_*i*_ and scores *S*_*i*_

2 $$U_{i} = a + b \, S_{i} + {\text{ res}}_{i}$$

Values, *V*, were calculated by deleting the residual, res_*i*_, i.e. *V*_*i*_ = *a* + *b* *S*_*i*_. Values calculated in this way are therefore a linear transformation of unweighted scores, *S*. Utilities, *U*_*i*_, determine the scale upon which values *V*_*i*_ are calibrated. Values differ from utilities by the ‘micro-utility effect’ included in res_*i*_.

### Removing scale effects

In each pairwise comparison of MAU_*i*_ and MAU_*j*_, the effect of scale was removed by rotating *U*_*j*_ and *V*_*j*_ to be on the same scale as *U*_*i*_. This was achieved by regressing *U*_*i*_ upon U_j_ and *V*_*j*_ as shown in Eqs. 3 and 4.

3 $$U_{i} = a_{1} + b_{1} U_{j} + {\text{ res}}_{ 1}$$

4 $$U_{i} = \, a_{2} + {\text{b}}_{ 2} V_{j} \, + {\text{ res}}_{ 2}$$

where res_1_ and res_2_ are residuals attributable to micro-utility effects and measurement error.

Rotated utilities and values for MAU_*j*_ were obtained from the linear component of these equations as defined by Eqs. 3′ and 4′.

3′ $$U_{j} (u_{i} )= a_{ 1} + b_{1} U_{j}$$

4′ $$V_{j} (u_{i} ) = a_{2} + b_{2} V_{j}$$

where *U*_*j*_(*u*_*i*_) and *V*_*j*_(*u*_*i*_) are, respectively, the utility and value from MAU_*j*_ rotated to be on the same scale as *U*_*i*_.

### Confirmation of result

The effect of the linear adjustment (3′) may be shown by substituting *U*_*j*_ = [*U*_*j*_(*u*_*i*_) − *a*_1_]/*b*_1_ derived from Eq. 3′ into 3.

$$U_{i} = a_{1} + b_{ 1} [U_{j} (u_{i} ) - a_{1} ]/b_{ 1} + {\text{ res}}_{ 1}$$

5 $$U_{j} \left( {u_{i} } \right) \, = \, U_{i} - {\text{ res}}_{ 1}$$

Similarly, substituting *V*_*j*_ = [*V*(*u*_*i*_) − *a*_2_]/*b*_2_ from Eq. 4′ into 4

$$U_{i} = a_{ 2} + b_{ 2} [V_{j} (u_{i} ) - a_{ 2} ]/b_{ 2} + {\text{ res}}$$

5′ $$V_{j} (u_{i} ) = U_{i} - {\text{ res}}_{ 2}$$

Equation 5 and 5′ confirm that in principle *U*_*j*_(*u*_*i*_) and *V*_*j*_(*u*_*i*_) are on the same linear scale as *U*_*i*_, varying from *U*_*i*_ by res_1_ and res_2_, respectively, which include the effects of differing descriptive systems, micro-utility effects and an error term. To test empirically the success with which scale effects were removed by these procedures, OLS regressions were estimated between differences in the scale-adjusted utilities and values: Eq. 6. With linear relationships between variables, a perfect alignment of scales would result in *a*_3_ = 0; *b*_3_ = 1.00. Nonlinearities in the relationships would result in *a* = 0 (a property of OLS regression) but possible deviation from *b*_3_ = 1.00.

6 $$[U_{i} - U_{j} (u_{i} )] = a_{ 3} + \, b_{ 3} [V_{i} (u_{i} ) - V_{j} (u_{i} )]$$

### Measuring the three components

**Disaggregation of the differences between utilities employed the following relationships:**
- * A* = *U*_*i*_ − *U*_*j*_: pairwise difference in utilities which are to be explained.
- *B* = *U*_*i*_ − *U*_*j*_ (*u*_*i*_): *‘scale-free’* *differences in utility.* The differences in utility measured on a common scale (MAU_*i*_).
- *C* = *A* − *B*: *the scale effect.* The amount of the difference, A, explained by measuring differences on a common scale.
- *D* = *V*_*i*_ − *V*_*j*_ (*u*_*i*_): *descriptive system effects.* The scale-free difference in values attributable (only) to differences in the descriptive system.
- * E* = *B* − *D*: *the micro-utility effect.* The scale-free differences in utility less the effect of differences in the descriptive systems.

### Combining the effects

$$\begin{aligned} {\text{Scale}}\, (C )&+ {\text{Descriptive}}\,\,{\text{system}}\, (D )+ {\text{micro}}\,\,{\text{utility}}\,(B - D) \hfill \\ \quad &= C + D + B - D \hfill \\ \quad &= C + B = (A - B) + B = U_{i} - U_{j} \hfill \\ \end{aligned}$$

## Results

### Data

Data were obtained from 9,665 individuals. Edit procedures resulted in the removal of 17 % of the total. Table 2 presents the age–gender and educational status of the remaining 8,019 respondents. Because quotas were imposed, the proportion of respondents from each country is similar. For the same reason, the age, gender and educational profiles of respondents within each country is similar. The numbers recruited from the disease area varied from 772 for cancer to 943 for heart disease. The 1,760 ‘public’ respondents were obtained by combining country samples which closely matched the age–gender profile in each country. There were few missing data as the online program did not permit respondents to proceed until questions were completed. Individuals who did not answer the final question were excluded. This resulted in a final sample of 8,019. A detailed comparison of utilities is given in Richardson et al. [5].

**Table 2:** Respondents Characteristics

Country	Composition of final sample																	
	Public (%)							Patient (%)							Education			
	18–24	25–34	35–44	45–54	55–64	65+	Male	18–24	25–34	35–44	45–54	55–64	65+	Male	High school	Diploma or certificate or trade	University	Total (*n*)
Australia	11.3	18.1	18.9	18.5	14.7	18.5	46.4	2.1	8.0	10.3	19.5	32.6	27.5	50.4	35.8	35.1	29.1	1,429
Canada	12.8	18.3	16.2	20.1	16.8	15.9	47.3	5.8	15.1	18.0	19.1	27.3	14.8	34.8	29.2	47.6	23.2	1,330
Germany	6.5	20.0	18.5	23.1	17.7	14.2	50.4	5.2	8.3	17.5	31.4	24.4	13.2	54.2	19.6	55.0	25.4	1268
Norway	12.8	16.0	16.7	18.4	15.6	20.5	50.3	6.2	8.2	10.2	16.8	26.0	32.6	63.6	28.0	48.5	23.5	1,177
UK	11.4	15.4	20.1	18.1	14.4	20.5	47.7	7.1	12.7	9.7	16.4	29.0	25.1	51.4	38.1	30.2	31.7	1,356
USA	10.3	17.8	18.1	20.2	16.2	17.4	45.2	4.8	8.8	13.1	25.0	25.5	22.8	36.4	36.1	29.3	34.6	1,459
Total	11.0	17.6	18.0	19.7	15.9	17.8	47.8	5.1	10.1	13.1	21.4	27.6	22.6	48.0	31.4	40.4	28.2	8,019

Table 3 reports summary statistics for the five instruments and the correlation between utilities and values. With the exception of the 15D mean utilities are similar, varying from 0.68 to 0.74 in the full sample and from 0.83 to 0.88 in the public sample. Despite this similarity, the distribution of utilities differ significantly. Reflecting scale differences, the standard deviation of the observations in the full sample varies by 100 % from 0.27 for HUI 3 to 0.13 for 15D and 0.14 for SF-6D. Ceiling effects (*U* = 1.00) vary from 19.1 % (EQ-5D) to 0.3 % (AQoL-8D), and the percentage with a utility below 0.4 varies from 0.3 for the 15D and 1.3 % for the SF-6D to 13.9 % for HUI 3 and 14.7 % for AQoL-8D. Values obtained from unweighted scores necessarily have the same means as utilities as they were obtained from the regression of utilities upon scores. However, as utilities are not a linear function of scores, the range of values differs from the range of utilities. Nevertheless, the correlation between values and utilities is very high, exceeding 0.89 in all cases and rising to 0.99 for the 15D.

**Table 3:** Summary statistics for the five MAU instruments (*n* = 8,019)

	Utility					Values			Correlation *ρ* (*U*, *V*)
	Mean	SD	Range	*U* = 1.00	*U* < 0.4	Mean	SD	Range	
EQ-5D	0.74	0.23	1.51	19.10	8.90	0.74	0.23	1.30	0.95
SF-6D	0.71	0.14	0.70	1.30	1.30	0.71	0.14	0.62	0.89
HUI 3	0.71	0.27	1.34	7.10	13.90	0.71	0.27	2.10	0.95
15D	0.85	0.13	0.75	6.90	0.30	0.85	0.13	0.67	0.99
AQoL-8D	0.68	0.22	0.90	0.30	14.70	0.68	0.22	1.32	0.98

### Rescaling

The linear regressions used to rotate the scales of utilities and values are reported in Table 4. The ‘b’ coefficient indicates the extent to which, on average, incremental change in the ‘independent’ (right-hand side) instrument utility or value must be compressed or expanded to be on the same scale as the ‘dependent’ (left-hand side) instrument. From the regression between HUI 3 and 15D utilities, increments of the 15D utility must be expanded by a factor of 1.75 for equivalence with the HUI 3 scale. In contrast, increments of utility on the AQoL-8D must be compressed by a factor of 0.47 for equivalence with incremental utilities measured by the 15D.

**Table 4:** GMS regression of *U*_*i*_ on *U*_*j*_ and *U*_*i*_ on *V*_*j*_ (*n*=8,019)

*U*_*i*_ = a + bU_j_ (Eq. 3)	*R* ^2^	*U*_*i*_ = a + bV_j_ (Eq. 4)	*R* ^2^
EQ-5D = −0.14 + 1.24 SF-6D	0.57	EQ-5D = −0.20 + 1.32 SF-6D	0.70
EQ-5D = 0.26 + 0.68 HUI 3	0.64	EQ-5D = 0.28 + 0.64 HUI 3	0.62
EQ-5D = −0.50 + 1.45 15D	0.67	EQ-5D = −0.50 + 1.46 15D	0.74
EQ-5D = 0.22 + 0.76 AQoL-8D	0.57	EQ-5D = 0.21 + 0.77 AQoL-8D	0.62
SF-6D = 0.44 + 0.37 HUI 3	0.53	SF-6D = 0.37 + 0.47 HUI 3	0.53
SF-6D = 0.0 + 0.81 15D	0.62	SF-6D = −0.02 + 0.86 15D	0.66
SF-6D = 0.37 + 0.49 AQoL-8D	0.65	SF-6D = 0.38 + 0.49 AQoL-8D	0.61
HUI 3 = −0.77 + 1.75 15D	0.70	HUI 3 = −0.78 + 1.76 15D	0.68
HUI 3 = 0.07 + 0.95 AQoL-8D	0.64	HUI 3 = 0.06 + 0.96 AQoL-8D	0.57
15D = 0.53 + 0.47 AQoL-8D	0.70	15D = 0.53 + 0.48 AQoL-8D	0.75

The test of the success of the rescaling of instruments is reported in Table 5. Reflecting the properties of the OLS regressions used to rotate the scales, a = 0 in every regression indicating that each of the variables used in the regressions has the same mean (equal to the mean of *U*_*i*_). In each case, the slope parameter, *b*, is close to but deviates from 1.00 reflecting nonlinearities in the relationship. In the disaggregation of effects, the imperfect alignment of scales will result in an increased micro-utility effect.

**Table 5:** Regression of scale-free difference between utilities and difference between values

MAU Pair		Regression^*^ *Y* = a + *bX*			MAU Pair		Regression^*^ *Y* = a + *bX*		
MAU_*i*_	MAU_*j*_	*a*	*b*	*R* ^2^	MAU_*i*_	MAU_*j*_	*a*	*b*	*R* ^2^
EQ-5D	SF-6D	0.00	0.83	0.52	SF-6D	15D	0.01	1.05	0.45
EQ-5D	HUI 3	0.00	0.97	0.64	SF-6D	AQoL-8D	0.00	0.94	0.48
EQ-5D	15D	0.00	1.12	0.61	HUI 3	15D	0.00	0.98	0.62
EQ-5D	AQoL-8D	0.00	1.06	0.69	HUI 3	AQoL-8D	0.00	0.92	0.69
SF-6D	HUI 3	0.00	1.00	0.50	15D	AQoL-8D	0.00	1.10	0.85

^*^ *Y* = [*U*_*i*_ − *U*_*j*_(*u*_*i*_)]; *X* = [*V*_*i*_(*u*_*i*_) − *V*_*j*_(*u*_*i*_)] n = 8,019

### Disaggregation

The decomposition of the pairwise differences in utilities is reported in Table 6. The average absolute difference between pairs of instrument utilities is 0.135. It varies from 0.114 (SF-6D, AQoL-8D) to 0.175 (15D, AQoL-8D). The largest component is the effect of the descriptive system which accounts for 66.0 % of the difference, varying from 27.4 % (15D, AQoL-8D) to 101.6 % (HUI 3, AQoL-8D). Scale affects average 30.3 % of the difference varying from 3.5 % (EQ-5D, SF-6D) to 69.7 % (15D, AQoL-8D). Micro-utility effects are the smallest component, averaging 3.7 % of the difference and the absolute value varying from 0.8 % (EQ-5D, HUI 3) to 19.8 % (EQ-5D, SF-6D).

**Table 6:** Decomposition of (*U*_*i*_ − *U*_*j*_)

Pairwise comparison^a^	Absolute differences				Per cent of (*U*_*i*_ − *U*_*j*_)			
	Utility (*U*_*i*_ − *U*_*j*_)	Scale-free diff in utility [*U*_*i*_ − U_j_(*u*_*i*_)]	Scale effect (*A* *−* *B*)	Descriptive system [*V*_*i*_ − *V*_*j*_(res)]	Micro utility (*B* *−* *D*)	Scale effect	Descriptive system	Micro utility
	*A*	*B*	*C*	*D*	*E*	(*C*/*A*)*100	(*D*/*A*)*100	(*E*/*A*)*100
EQ, SF	0.116	0.112	0.004	0.089	0.023	3.5	76.72	19.8
EQ, HUI	0.117	0.101	0.016	0.101	0.001	13.7	85.5	0.8
EQ, 15D	0.130	0.097	0.033	0.083	0.013	25.7	64.3	10.0
EQ, AQoL	0.130	0.112	0.018	0.105	0.007	13.9	80.8	5.3
SF, HUI	0.146	0.078	0.069	0.075	0.003	47.0	50.9	2.1
SF, 15D	0.144	0.069	0.075	0.062	0.007	52.1	43.0	4.9
SF, AQoL	0.114	0.065	0.049	0.067	−0.002	43.0	58.8	−1.8
HUI, 15D	0.154	0.108	0.046	0.110	−0.002	29.9	71.4	−1.30
HUI, AQoL	0.125	0.120	0.005	0.127	−0.007	4.0	101.6	−5.60
15D, AQoL	0.175	0.053	0.122	0.048	0.005	69.7	27.4	2.9
Average	0.135	0.092	0.043	0.085	0.007^b^	30.3	66.0	3.7

^a^EQ=EQ-5D-5L; SF=SF-6D; HUI = HUI 3; AQoL =AQoL-8D

^b^Average of absolute values

## Discussion

Discrepancies between utilities predicted by different MAU instruments have been observed in a very large number of studies [1]. Consistent with these, the present study also identifies quantitatively large differences. Across all pairwise comparisons, the average difference in utilities predicted for the 8,019 survey respondents was 0.135. To put this figure in perspective, an incremental change in utility of 0.135 for seven people is almost equivalent to the difference between death and full health for a single person: that is, the difference is quantitatively large with correspondingly large implications for the outcome of an economic evaluation.

The chief conclusion from the present study is that these differences are primarily the result of differences in the descriptive systems. While these explain an average of 66.0 % of the difference between utilities, their importance in pairwise comparisons varies from 27.4 % in the comparison of the 15D and AQoL-8D to 101.6 % of the difference between HUI 3 and AQoL-8D. The former results are plausible. As scale effects account for a larger part of the difference between 15D and AQoL-8D than for any other instrument pair, the relative importance of the remaining effects is consequently reduced. In Table 1, the 15D descriptive system uniquely shares with AQoL-8D items relating to sleep and intimacy and the two instruments have the largest number of items describing depression and anxiety. In contrast, the ‘within the skin’ descriptive system of HUI 3 has no items relating to social relationships which constitute a major part of the AQoL-8D descriptive system.

The more surprising result is that the principle effect of differing utility weights is via their effect upon measurement scales and not upon the micro-utility effect. The scale effects are large in comparisons involving 15D, and from Table 3, the 15D has the lowest standard deviation implying the greatest compression of utilities. Scale effects are also large in the comparison of SF-6D with both HUI 3 and AQoL-8D. From Table 3, the SF-6D has the second lowest standard deviation and the HUI 3 and AQoL-8D have the largest standard deviations.

After taking account of differences in the descriptive system and scale, the residual micro-utility effect is generally positive: the effect contributes to an explanation of differences. In three cases in Table 6, it is negative suggesting that the effect partially compensates for other differences. With one exception, the effect is small. The exception is the estimated micro-utility effects in the comparison of EQ-5D and SF-6D. From Table 3, the relationship between SF-6D and EQ-5D is particularly nonlinear with a rapid decrease in SF-6D utilities at the top end of the scale where 19 % of EQ-5D utilities but only 1.3 % of SF-6D are equal to 1.00. The pattern reverses as health deteriorates with 1.3 and 8.9 % of observations below 0.4 for the SF-6D and EQ-5D, respectively. Using present methods, the effect of nonlinearities in the relationship between utilities is attributed to the micro-utility effect.

The respective magnitudes of the three effects employed in the disaggregation have implications for the practice and future development of CUA. First, the identification of significant scale effects implies that these should be eliminated by mapping utilities to a common scale in any ranking of interventions which have employed different MAUI. Mapping functions between each pair of instruments have been estimated by Chen et al. [16] from the database used in the present study and are available on the AQoL website.

Secondly, the results call into question the usefulness of past and future research which is justified by the need to incorporate particular preferences. Unique preferences in Australia, Canada, Finland and the UK would have resulted in significant micro-utility effects in the comparison of the MAUI which derived utilities from representative samples in those countries. The small effects found here suggest that differences in utilities attributed to national preferences are probably the result of differences in the methodologies used to derive utility formula. Minimally, before new results can be attributed to unique preferences the effects of the methods upon utilities must be taken into account.

Finally, as the differences between utilities were primarily attributable to differences in the instrument’s descriptive systems, these differences will not be fully eliminated by mapping to a common scale or by the re-estimation of utilities. This implies that the results of a CUA may depend upon the choice of MAUI. Elsewhere, we argue that the most sensitive instrument in a disease area should be selected and utilities transformed to the scale of a single instrument [5]. The comparison of results from different instruments will remain imperfect but will be superior to the use of a single instrument which is more sensitive to some health states than to others.

A caveat to the present results is that the effect of measurement error—the inconsistent and erroneous completion of two questionnaires—will result in a larger apparent effect of the descriptive systems. The problem is difficult to circumvent as survey respondents are fallible. However, it is unlikely to have had a large impact. The MIC data were subjected to eight separate edit procedures to delete inconsistent results. These were based upon the comparison of repeated and similar questions and resulted in the removal of 17 % of respondents from the database before analyses commenced. Remaining inconsistencies are unlikely to explain the magnitude of the effects identified here. A more plausible explanation is that the effect is a correct reflection of the very significant differences in the descriptive systems which are apparent from the casual comparison of the instruments.

A final caveat to the results is that they are necessarily based upon particular published utility formulae. While the effect of the descriptive systems is independent of the utility weighting, both the scale and micro-utility effects could vary substantially with a change in the utility formula.

## Conclusions

The validity of CUA is compromised by the inconsistent results of the MAUI used to estimate QALYs. A significant body of research has sought to increase the validity of utility measurement by refining the methods used for eliciting utilities, or by deriving utilities from nationally representative samples. The present paper has investigated the extent to which such research is likely to reconcile the inconsistencies in the MAUI. The results suggest that utility weights are important, accounting for 34 % of the difference between instrument scores. But their impact is primarily via a scale effect: different utility formula use different scales for the calibration of utility and these account for 30.3 of the 34.0 % difference between utilities attributable to utility weights. It is possible that this result is attributable to differences in the modelling methodologies that have been adopted. After adjusting for this, the residual effect of different formula—the ‘micro-utility effect’—is relatively small. This implies that there is little scope for reconciling the numerical values obtained from different instruments by achieving greater precision in the relative values assigned to items.

The dominant determinant of the difference between utilities is the difference between descriptive systems. A necessary condition for achieving comparability between utilities, QALYs and, therefore, the results of cost-utility analyses is the use of instruments with comparable descriptive systems or the adjustment of results to take account of structural and scale differences.

## Appendix

See Table 7.

**Table 7:** OLS regression of *U*_*i*_ on score_i_

MAU_i_	*a*	*b*	*R* ^2^
EQ-5D	−0.38	1.33	0.90
SF-6D	0.21	0.71	0.80
HUI 3	−1.53	2.54	0.95
15D	−0.02	1.02	0.98
AQoL-8D	−0.36	1.44	0.96

*U* = *a* + *b* Score (*n*=8,019)
